# Effect of Sodium Treatment on the Performance of Electrostatic Spray Assisted Vapour Deposited Copper-poor Cu(In,Ga)(S,Se)_**2**_ Solar Cells

**DOI:** 10.1038/s41598-017-07027-9

**Published:** 2017-07-28

**Authors:** Mingqing Wang, Md. Anower Hossain, Kwang-Leong Choy

**Affiliations:** UCL Institute for Materials Discovery, University College London, Roberts Building, Malet Place, London, WC1E 7JE United Kingdom

## Abstract

In our work, eco-friendly, non-vacuum and low cost Electrostatic Spray Assisted Vapour Deposition (ESAVD) method has been used to produce Cu(In,Ga)(S,Se)_2_ (CIGS) solar cells. Copper (Cu) deficient (Cu/In + Ga = 0.76) CIGS films were designed to avoid the rather dangerous KCN treatment step for the removal of conductive minor phases of Cu_2_S/Cu_2_Se. A simple sodium (Na) treatment method was used to modify the morphology and electronic properties of the absorber and it clearly improved the solar cell performance. The SEM and XRD results testified a slightly increase of the grain size and (112) crystal orientation in the Na-incorporated CIGS thin films. From the Mott-schottky results, it can be seen that the functions of the Na treatment in our non-vacuum deposited CIGS are mainly used for defect passivation and reduction of charge recombination. Photovoltaic characteristics and *j-V* curve demonstrated that the dipping of CIGS films in 0.2 M NaCl solution for 20 minutes followed by selenization at 550 °C under selenium vapor resulted in the optimum photovoltaic performance, with *j*_sc_, *V*_oc_, *FF* and *η* of the optimized solar cell of 29.30 mA cm^−2^, 0.564 V, 65.59% and 10.83%, respectively.

## Introduction

Chalcopyrite Cu(In,Ga)Se_2_ (CIGS) solar cells have shown great potential in thin film photovoltaics technologies, with record solar cell efficiencies of 22.3% and commercial solar modules with efficiencies in the range of 12–14%^1, 2^. In addition to the high efficiency, the CIGS solar cells also exhibit advantages, such as better resistance to heat and radiation as compared to silicon-based solar cells, excellent long-term stability than organic solar cells or organic/inorganic hybrid solar cells^3, 4^, and it is believed to be one of the most promising commercial solar modules. However, market share of CIGS devices have been limited due to the inherent high cost and low deposition rate of the physical vapour deposition (PVD) techniques employed in the manufacturing flagship programs. The conventional PVD deposited CIGS films accounts for up to 40% of the total device cost. In order to make the CIGS modules-based solar industry much more competitive and sustainable in the long-run, the CIGS thin film solar technologies using low cost and non-vacuum processes, such as electrodeposition^5^, hydrazine^6^, quantum dots^7^, and other wet-chemical precursor-based direct liquid coating of metal nanoparticles^8^, metal salts^9^, metal sulphides^10^ and metal oxides^11^ have been developed^12^. Electrodeposition (ED) is a non-vacuum method that has been studied for the deposition of CIGS thin films since the early 1980’s^13^. The widely used ED approach includes two steps, electroplating of metallic Cu-In and/or Cu-In-Ga precursors followed by selenization at temperatures above 500 °C to form semiconducting quality CIGS films. While the efficiency of the ED-based solar cells have achieved 15.4%^10^, a post-composition adjustment deposited by physical vapour deposition approach is often required to obtain Cu-poor CIGS films. Hydrazine-based methods are another approach to improve the compositional control and quality of the solution-processed CIGS thin films. Hydrazine is an extremely polar liquid with unique reducing nature, which can dissolve certain metal selenides and sulfides at high concentrations with extra chalcogens. CIGS solar cells based on hydrazine method have achieved efficiency of 15.2%^14^. However, hydrazine is highly toxic and flammable, making it difficult to transfer this technique to the large-scale industrial production. Other organic solvents which are less toxic and also can form metal-organic complexes (such as 1,2-propanediol^15^, carbon disulphide^16^, and dimethyl sulfoxide^17^) have been developed to replace the very toxic hydrazine for CIGS absorbers. These alternative solvents are less toxic as compared to hydrazine, while they are still hazardous in case of contact and are not environmentally friendly. Hot injection is one of the popular methods to make CIGS nanoparticles. The synthesized nanoparticles can be dispersed in various non-polar solvents such as hexanethiol, toluene *etc*. to form a stable ink for subsequent printing onto various substrates^7, 18^. After the subsequent post-selenization and KCN treatment of the deposited CIGS thin films, solar cells based on hot injection method demonstrate a maximum energy conversion efficiency of 15%^19^. However, disadvantage of the hot injection method is that it requires complex synthesis and purification procedures, and CIGS quantum dots are normally synthesized under protection gas in small-scale research labs.

Non-vacuum Electrostatic Spray Assisted Vapour Deposition (ESAVD) has been proven to be suitable for its low cost, environmental friendly precursors, and non-vacuum deposition method of metal oxides, different sulphides and chalcogenides in air^20^. During ESAVD process, a mixture of chemical precursors is atomized to form an aerosol. The aerosol is charged and directed towards a heated substrate where it would undergo decomposition and chemical reaction to form a stable solid film onto the substrate. The use of an electric field during the deposition helps to direct the chemical precursor toward the well-defined area of the heated substrate, thus enabling high deposition efficiency (*e.g*., above 90%) at optimum deposition conditions. Such high deposition efficiency of the ESAVD process is especially valuable for CIGS films, for which the indium (In) and gallium (Ga) constituents are expensive and the elemental content in the earth is comparatively low. In addition, as compared with the spin-coating method which is widely used in the other published works, the ESAVD process can easily be adapted to large area deposition using multiple spray atomizers, which is crucial for the scale up of the non-vacuum CIGS solar modules. In our previous work, the ESAVD method has been adapted for deposition of the CIGS absorber^21^. The use of environmentally friendly solvents is also an advantage of the ESAVD process. Thiourea (CH_4_N_2_S) is selected to complex with metal ions and act as a sulphur (S) source to form CIGS once the precursor approaches the hot substrate. By optimizing deposition conditions and composition in the precursor, CIGS solar cells with efficiency above 8% have been achieved by the ESAVD method^22^.

In order to achieve CIGS solar cells with a high efficiency, micron-sized grains is normally required to suppress the recombination of photo-generated charge carriers at grain boundaries. Quasi-liquid phase Cu_2−x_Se formed in Cu rich absorber usually serves as a flux agent to enhance the crystallization of CIGS^23^. However, an extra KCN treatment step is required to remove secondary phases of Cu_2−x_Se/Cu_2−x_S, which may shunt the solar cells because of its comparatively high electric conductivity^24^. In the ESAVD process, the CIGS films were intentionally prepared with slightly Cu-poor condition(Cu/In + Ga = 0.76) than usual(Cu/In + Ga > 0.80) and the Cu content in the precursor solution was adjusted accordingly, which eliminates the formation of Cu_2−x_Se and avoids the use of the dangerous KCN post-treatment step. It has been proven that the incorporation of Na into the CIGS absorber could lead to a significant enhancement of device performances in the vacuum processed CIGS solar cells. The main improvement after Na incorporation includes the enhancement of crystal structure, electronic characteristics and photovoltaic properties: (i) an increase in grain size and a strong (112) orientation of the CIGS film^25^; (ii) an increase in the hole concentration and *p*-type conductivity due to defect passivation^26^; and (iii) increased open circuit-voltage (*V*_oc_) due to the enhanced p-type semiconducting characteristics^27^. In addition, there are also reports on the increase in short circuit current (*j*_sc_) and fill factor (FF) of devices after Na doping^28^. It is noteworthy to mention that if the CIGS absorber becomes more Cu-poor, more dramatic improvement of the Na incorporation on CIGS solar cells can be achieved^29^. This would suit our case, to deliberately deposit Cu-poor absorber layers in order to avoid the highly toxic KCN treatment. The Na can be incorporated into CIGS film by the diffusion from soda-lime glass (SLG) glass or by physical vapour deposition of an additional NaF layer. In our previous work^30^, in order to make the Na doping process more compatible with our non-vacuum deposition process for the absorber, Na doping was achieved by directly dipping the CZTS in aqueous NaCl solution followed by high temperature selenization under Se vapor. NaCl is chosen as the Na source because of its high solubility, low cost and abundance in nature. In the following work, a similar process was adopted for Na treatment of CIGS absorber.

## Experiment and characterization

### CIGS deposition using ESAVD and selenization

The CIGS absorber films were deposited at substrate temperatures ranging from 250–450 °C from a mixture of chemical precursor solution containing the salts of Cu, In, Ga, and thiourea. After deposition, CIGS absorbers were dipped into NaCl aqueous solutions with different concentrations for 20 minutes. The CIGS films were subsequently selenised in an argon filled tube furnace at 550 °C for 30 minutes. After selenization, the as-deposited CIGS films was converted into Cu(In,Ga)(S,Se)_2_ (CIGSSe), in which various defect states at grain boundary regions were expected to be suppressed. Subsequently, they were cooled down to room temperature and immediately transferred into a chemical bath for CdS deposition.

### Composition characterization

Composition of the as-deposited CIGS films was characterized by X-ray fluorescence (XRF, Fischerscope X-Ray Xan250). After selenization, CIGS absorber was characterized by Energy Dispersive X-Ray spectroscopy (EDX).

### Structural characterization

Powder X-ray diffraction (XRD) patterns were collected using a Bruker D8 Discover Diffractometer. A Renishaw Raman microscope with a tunable Argon ion laser at 514 nm was used to collect the Raman spectra. The surface morphology and cross-section images were taken using Scanning Electron Microscope (SEM, JEOL JSM-6700F) and JEOL 6480LV SEM, respectively.

### Fabrication and characterization of solar cells

The most common device structure for CIGS solar cells (soda-lime glass/Mo/ CIGSSe/CdS/i-ZnO/AZO/Ni/Al) was used in our work. Chemical bath deposition was used to deposit a 50-nm-thick CdS buffer layer, and RF sputter (HHV) was used to sequentially deposit circa. a 50-nm-thick intrinsic ZnO layer and a 700 nm thick Al-doped ZnO layer as a window layer for CIGS solar cells. Thermal evaporation was subsequently used to deposit a 200-nm-thick Ni/Al patterned collection grids on top of the device as the finger electrode. The dimension of the individual solar cell was scribed and defined circa. 0.15 cm^2^. A solar simulator (Oriel Sol 1 A) was utilized to measure the photovoltaic (*j-V*) characteristics of solar cells under the simulated AM 1.5 100 mW cm^−2^ illumination. External quantum efficiency (EQE) spectra were obtained using a Spequest qunatum efficiency photovoltaic system (Rera) equipped with a Xenon/quartz lamp. The incident photon flux was determined using the calibrated silicon (Si) and germanium (Ge) photodiodes, which allowed us to measure the EQE up to a photon wavelength of 1400 nm.

### Electrochemical Test

The electrochemical experiments were carried out using an Autolab electrochemical workstation. These involved the use of 0.1 M tetrabutylammonium hexafluorophosphate (TBAPF_6_) acetonitrile (MeCN) solution in a three-electrode configuration under a dark environment. Pt wire, Ag/AgCl electrode and CIGS films were selected as counter electrode, reference electrode, and working electrode, respectively.

## Results and Discussion

### Compositional study by XRF and EDX

Compositional results of the as-deposited CIGS films used for Na treatment was measured by XRF which is shown in Table 1. The calculated values of Cu/(In + Ga) ratio and the Ga/(In + Ga) ratio are 0.67 and 0.23 respectively. The Cu/(In + Ga) ratio was intentionally controlled to be lower than the widely used ratio of 0.8 considering the In loss and composition change during selenization process. Six CIGS samples were deposited under similar conditions. One was used as a reference without any treatment, and the remaining five samples were treated with 0.1 M, 0.2 M, 0.3 M, 0.4 M and 0.5 M NaCl aqueous solutions, respectively, in order to establish the Na treatment effect.

**Table 1:** Compositional study of the as-deposited CIGS films by XRF.

CIGS Samples	Cu (at%)	In (at%)	Ga (at%)	S (at%)	Cu/(In + Ga)	Ga/(In + Ga)
As-deposited	20.56	23.52	7.12	50.4	0.67	0.23

The composition of selenized CIGS films were characterized by EDS, and the results are summarized in Table 2. As shown in Table 2, both the ratios of Cu/(In + Ga) and Ga/(In + Ga) of CIGS absorbers increased understandably to 0.75–0.76 and 0.24 respectively after selenization. This should be due to the loss of In during selenization process. While there is almost no difference found between the chemical composition ratios of the six CIGSSe films for comparison of Na treatment. The Cu/(In + Ga) ratio of 0.75–0.76 is indicative of the feasibility of omitting the toxic KCN treatment.

**Table 2:** Compositional study of the selenized CIGSSe films by EDS elemental analysis.

CIGSSe Samples	Cu (at%)	In (at%)	Ga (at%)	S (at%)	Se (at%)	Cu/(In + Ga)	Ga/(In + Ga)
0.1 M NaCl	16.97	17.02	5.45	11.98	48.58	0.76	0.24
0.2 M NaCl	16.72	16.52	5.29	12.85	48.62	0.76	0.24
0.3 M NaCl	16.92	17.12	5.43	12.33	48.12	0.75	0.24
0.4 M NaCl	17.08	17.20	5.36	11.92	48.44	0.76	0.24
0.5 M NaCl	17.02	16.94	5.35	12.41	48.28	0.76	0.24
0 M NaCl	17.73	17.72	5.68	10.65	48.22	0.76	0.24

### X-Ray diffraction

**Figure 1:**
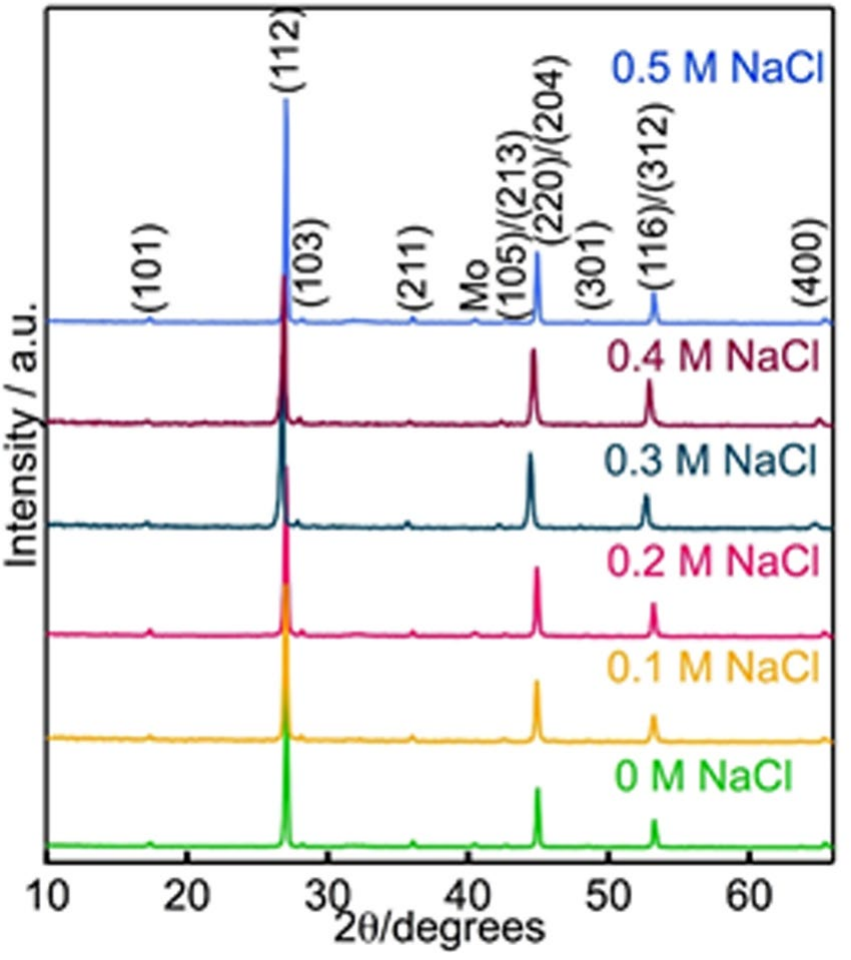
XRD patterns of CIGSSe absorbers with and without Na treatment.

The XRD patterns of the selenised CIGSSe thin films with and without NaCl treatment under various conditions are shown in Fig. 1. The main XRD peaks at 2θ of 28.3°, 46.9° and 53.0° of the as deposited absorbers belong to (112), (204)/(220) and (116)/(312) orientations of polycrystalline chalcopyrite CIGS structure, respectively, which indicated that polycrystalline CIGS films were successfully formed without the co-existence of other undesired binary or ternary phases. The films show similar characteristic diffraction peaks of the CIGSSe films for all samples, which showed (112) dominant orientation. The maximum peak intensity ratio of I_1 1 2_/I_2 2 0/2 0 4_ increased with the Na treatment concentration, which is consistent with the results reported elsewhere in the literature^18^.

### SEM

**Figure 2:**
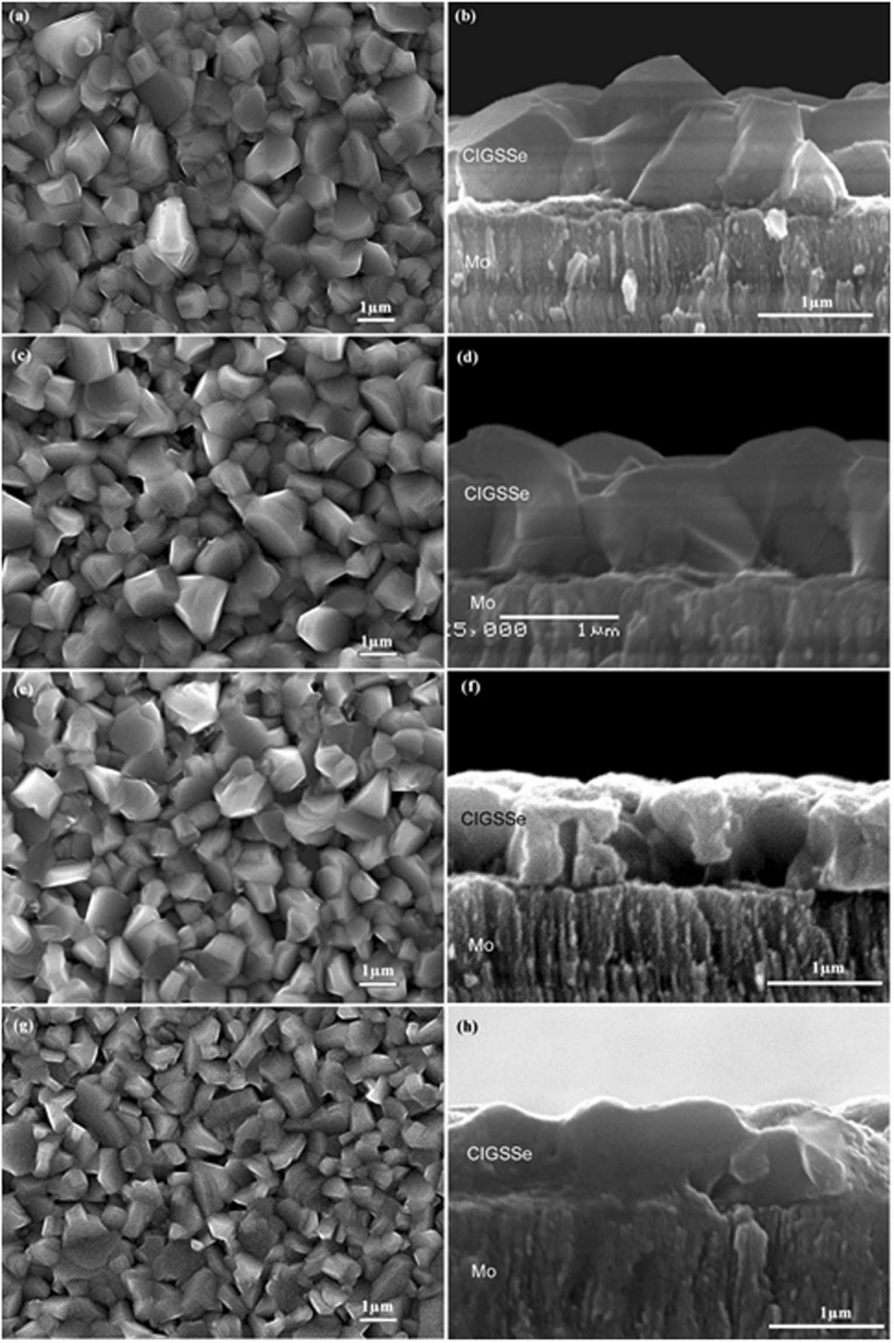
SEM top-view and cross-sectional images of the selenized CIGSSe film treated with (**a,b**) 0.2 M NaCl, (**c,d**) 0.5 M NaCl, (**e,f**) 0.1 M NaCl aqueous solutions for 20 minutes, and (**g,h**) untreated CIGSSe samples.

The scanning electron micrographsof the CIGSSe thin films with and without Na doping are shown in Fig. 2 (SEM top-view and cross-sectional images of the selenized CIGSSe film treated with 0.3 M NaCl and 0.4 M NaCl aqueous solutions were demonstrated in supporting information.). It can be seen from SEM images of the top and cross-sectional of CIGSSe thin films that the average grain size was increased slightly with the increase in Na treatment concentration. After Na doping, the average crystal grains size of CIGS absorber was circa. one micron, whereas the untreated CIGSSe showed comparatively smaller crystal grains, suggesting that the Na treatment helps improving the crystallinity of the CIGSSe films. The increased grain size of Na treated CIGS absorber would decrease the charge recombination at the grain boundaries^31^.

### Raman spectroscopy

**Figure 3:**
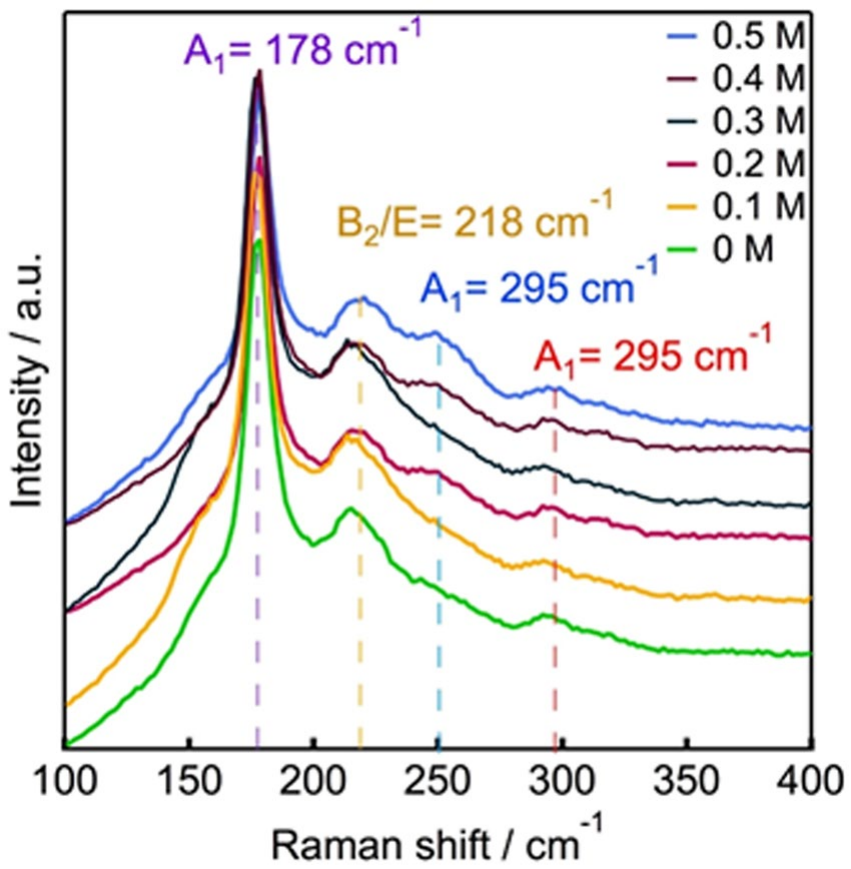
Raman shift of CIGSSe thin film absorbers with and without Na treatment.

In XRD patterns, the peaks of CIGS almost correspond to that of Cu_2_Se, so it is difficult to investigate single phase from XRD measurement. Raman analysis is able to detect different phases, which may not be distinguishable by X-ray diffraction techniques through their characteristic scattering peaks^32^.Therefore, Raman characterisation was performed to determine the presence of any secondary phases and to verify the purity of chalcopyrite CIGSSe thin films. As it can be seen from Fig. 3, CIGSSe thin films show two strong characteristic peaks of CIGSSe. The peak at 178 cm^−1^, associated with the “A_1_” mode of lattice vibration for the chalcopyrite structures, and another peak at 218 cm^−1^, associated with the “B_2_/E” mode of lattice vibration for the chalcopyrite structures. The peak at 295 cm^−1^ is associated with the “A_1_” mode of CIGS in the absorber due to the existence of a small amount of S remaining in the absorber during selenization. Moreover, due to the low Cu content, a small shoulder at around 150 cm^−1^ assigned to the formation of ordered defect compounds is also shown but it is not clearly recognizable. The absence of other Raman peaks indicates the phase pure CIGS films.

### Current-voltage (*j-V*) characteristics

**Figure 4:**
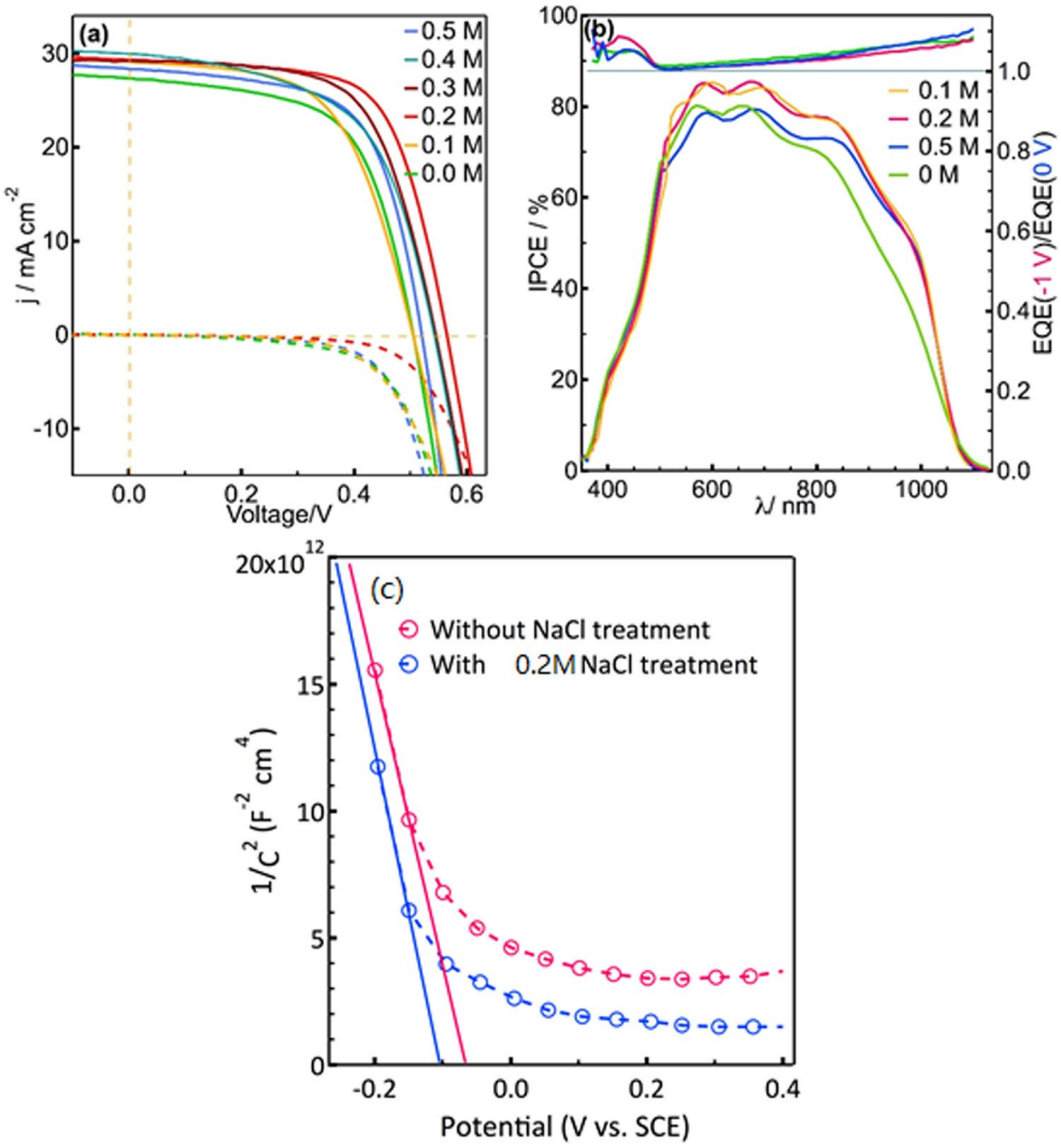
(**a**) j-V characteristics of solar cells measured under simulated AM 1.5 100 mW cm^−2^ illuminations, (**b**) EQE and (**c**) Mott-Schottky of the CIGSSe thin film solar cells with and without Na treatment.

In order to investigate the effect of Na doping on the photovoltaic performance, CIGSSe solar cells with the common used configuration (*i.e*. soda-lime glass/Mo/CIGS/CdS(50 nm)/i-ZnO(50 nm)/AZO(400 nm)/Ni(50 nm):Al(200 nm)) were fabricated. Photovoltaic parameters of the devices with and without Na treatment are shown in Table 3. The related *j-V* curves and External Quantum Efficiency (EQE) characterization are shown in Fig. 4. The reference solar cell without Na treatment exhibited *j*_sc_ of 27.26 mA cm^−2^, *V*_oc_ of 0.505 V, *FF* of 60.73% and efficiency (*η)* of 8.36%, respectively. Na treatment in NaCl solution before selenization clearly improved the device performance. The optimum Na treatment condition is dipping the CIGS absorber in 0.2 M aquesous NaCl solution for 20 min, which leads to an increase in all of the photovoltaic parameters of *j*_sc_, *V*_oc_, *FF* and *η* to 29.30 mA cm^−2^, 0.564 V, 65.59% and 10.83%, respectively. With the increase of the concentration of NaCl from 0 to 0.5 M, the efficiency of CIGS solar cells increased from 8.36% (0 M NaCl) to 10.83% (0.2 M NaCl), and then dropped to 9.26% (0.5 M NaCl).

**Table 3:** *Photovoltaic parameters* of CIGSSe solar cells under simulated AM 1.5 G, 100 mW cm^−2^ illumination.

*Solar cells’ Condition*	*V*_oc_ (V)	*j*_*sc*_ (mA cm^−2^)	*FF* (%)	*η* (%)	*R*_*s*_ (Ω cm^2^)	R_*sh*_ (Ωcm^2^)
0.0 M NaCl	0.505	27.26	60.73	8.36	3.16	217.54
0.1 M NaCl	0.504	29.02	58.05	8.49	2. 84	227.86
0.2 M NaCl	0.564	29.30	65.59	10.83	2.88	374.51
0.3 M NaCl	0.550	29.24	62.49	10.05	2.92	327.44
0.4 M NaCl	0.542	29.92	58.78	9.37	2.79	221.45
0.5 M NaCl	0.520	28.23	63.06	9.26	2.66	247.76

### External quantum efficiency

EQE measurements, as depicted in Fig. 4(b), show conversion of photons of CIGS solar cells beyond 1100 nm in the solar spectrum. The highest photocurrent response for the CIGSSe solar cells with 0.2 M NaCl dipping for 20 minutes was observed to be circa. 85%. The CIGSSe solar cells with 0.2 M NaCl dipping treatment showed improved photo response in the long wavelength region near the band edge; this indicates improved carrier collection efficiency due to a reduction of charge recombination loss deep in the CIGSSe layer, or a longer minority carrier diffusion length. The EQE improvement is consistent with the increase of *j*_sc_ obtained from the *j-V* test. Both *j-V* curve and EQE figures showed that the incorporation of Na with higher concentration (0.5 M NaCl for 20 minutes) would degrade solar cell performance because of the introduction of additional deep defect states.

The EQE bias ratio, [EQE(−1 V)/EQE(0 V)] (ratio of EQE measured at −1 V and 0 V) was also plotted in order to study the principle of the improvement of photo response of 0.2 M Na treated device. As shown in the top section of Fig. 4(b), an increasing [EQE(−1 V)/EQE(0 V)] ratio with photon wavelength-especially at longer wavelength, suggests a voltage-dependent collection efficiency which occurs in all of the solar cells with short minority carrier diffusion lengths^33^. As compared with the reference device without Na treatment and the device with 0.5 M Na treatment, CIGS solar cell with 0.2 M NaCl treatment showed a decreased [EQE(−1 V)/EQE(0 V)] ratio at the long wavelength. This further proofs the increase of effective charge separation between electron−hole pairs of the 0.2 M NaCl treated CIGS solar cells.

### Mott-Schottky Plots

To verify the free carrier density, Hall measurements of selenized CIGS films on SLG glass without and with Na treatment were performed. Because of the low mobility and high sheet resistance of the low Cu content ESAVD deposited CIGS thin films, bulk carrier concentration, mobility and Hall coefficient values were not stable. In order to better understand the effect of Na doping on the electrical properties of CIGS absorber films, Mott-Schottky (MS) was adopted to investigate the carrier concentrations in the CIGS thin films using Electrochemical Impedance Spectroscopy(EIS). The CIGS absorbers treated in 0 M and 0.2 M NaCl solution were used for comparing the effect of Na treatment on the electrical properties of CIGS absorber. Figure 4(c) shows MS plots (1/C^2^ vs. potential, where C is the capacitance of the semiconductor) of CIGS absorbers. The slope of the MS plots was found to be negative, confirming that the CIGS films are p-type. The flat band potential and carrier concentration of the CIGS films can be calculated from the MS analysis using the following equation^34^:

1 $$\frac{1}{{C}^{2}}=\frac{-2}{\varepsilon {\varepsilon }_{0}e{N}_{A}{A}^{2}}(V+{V}_{fb}+\frac{kT}{e})$$

where, C is the capacitance, ɛ_0_ is the permittivity of vacuum, ε is the dielectric constant of CIGS, *e* is the electric charge, N_A_ is the acceptor concentration, A is the active area, V is the applied potential against Ag/AgCl electrode, k is the Boltzmann constant, T is the absolute temperature, and V_fb_ is the flat band potential. The carrier concentration obtained from MS curve is 2.11 × 10^17^ cm^−3^ and 7.48 × 10^17^ cm^−3^ for CIGS film with and without 0.2 M Na treatment, respectively. The obtained carrier concentration is higher than the value (the order of 16 per cm^3^) obtained in commercially vacuum deposited CIGS absorber as previously reported by Friedlmerier *et al*.^35^. Contrary to the Na doping effect on many vacuum deposited CIGS absorber, extra Na treatment on our non-vacuum deposited CIGS decreased instead of increased the carrier concentration in p-type CIGS. Based on the above results, we proposed a new Na treatment principle. In our work, Na can be incorporated into the CIGS absorber through the diffusion from SLG glass during selenization process. The main function of extra Na treatment is to passivate the defect at interface and around grain boundaries.

The depletion width (W_d_) of *p-n* junction at zero bias in CIGS devices can be determined using the following equation^36^:

2 $${W}_{d}=\frac{\varepsilon {\varepsilon }_{0}}{C/A}$$

Combining equation (1) and (2), it can be concluded that CIGS device with low carrier density generally shows a low capacity and wide depletion width. The wider depletion width could enhance the collection of photogenerated charge carriers, especially if the minority carrier diffusion length is small (non-vacuum deposited absorber).

### Efficiency distribution

**Figure 5:**
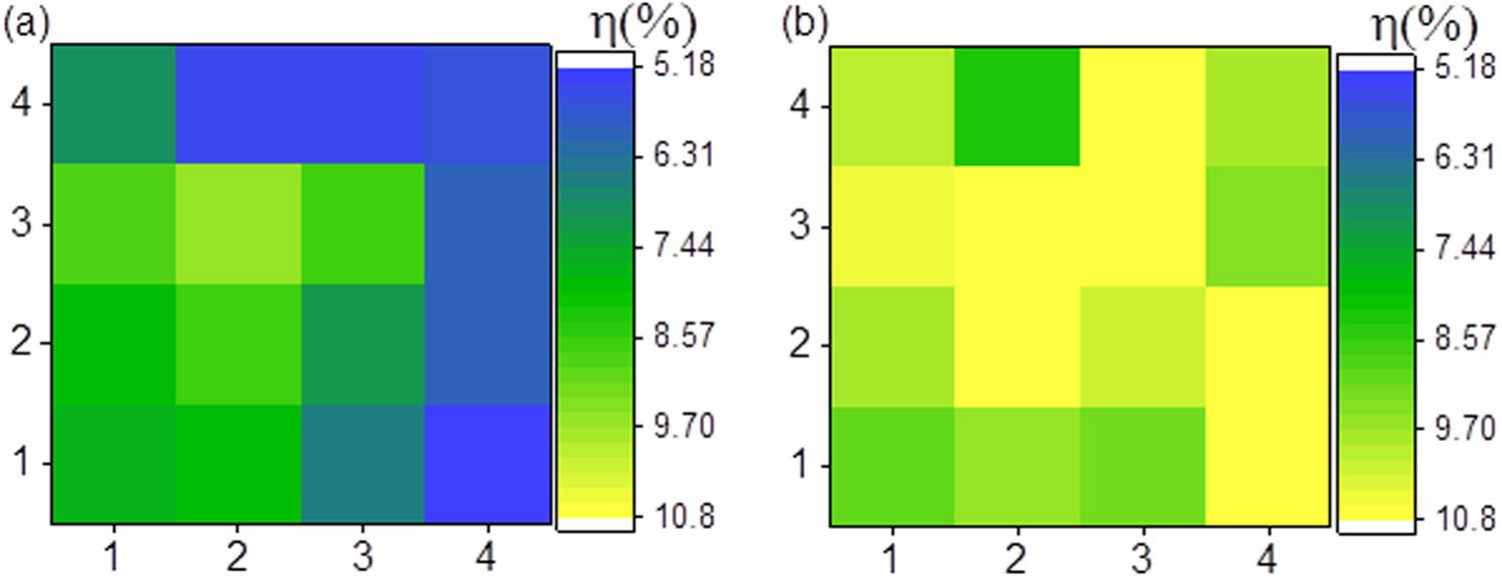
Efficiency distribution of ESAVD deposited CIGSSe solar cells: (**a**) without NaCl treatment and (**b**) with 0.2 M NaCl extra Na post-treatment.

The efficiency distribution of ESAVD deposited CIGS solar cells with and without extra Na treatment is shown in Fig. 5. Without extra Na treatment, the solar cell devices shows average efficiency of 7.13% with standard deviation of 1.38%. After Na treatment in 0.2 M NaCl aqueous solution, not only average value but also efficiency distribution of the solar cells were greatly improved (with average efficiency of 10.02% with standard deviation of 0.76%).

### Cross-sectional SEM of the device

**Figure 6:**
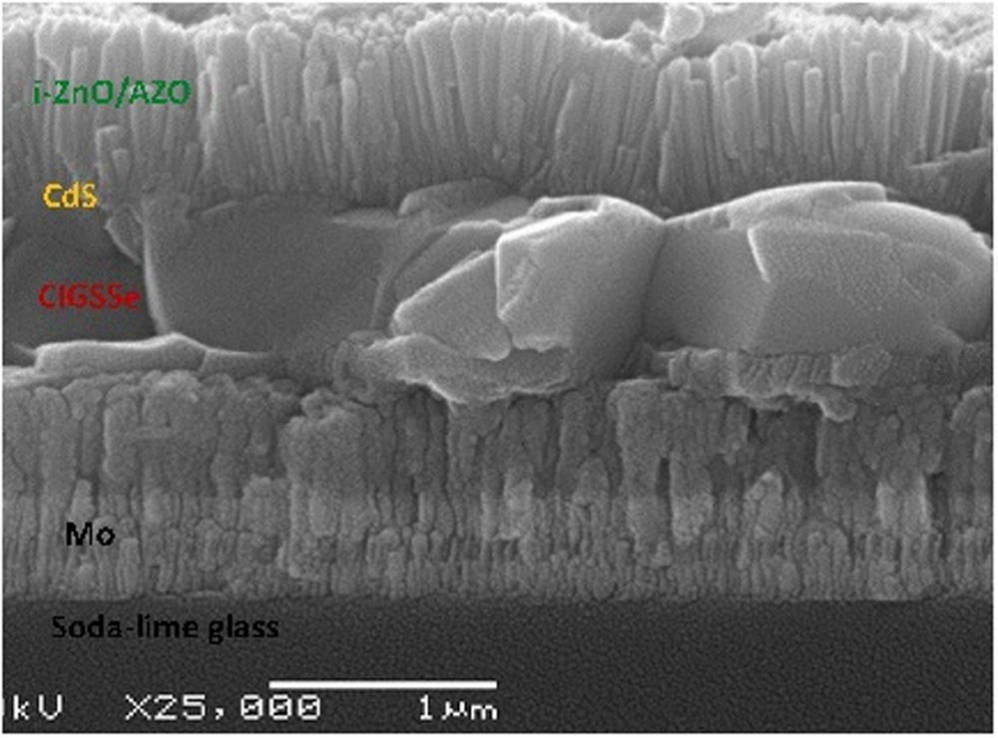
Cross-section SEM image of the champion CIGS solar cells with 10.83% efficiency (dipping in 0.2 M NaCl aqueous solution for 20 minutes).

Cross-sectional SEM image of the champion CIGS solar cells with 10.83% efficiency (dipping in 0.2 M NaCl aqueous solution for 20 minutes) is further characterized and shown in Fig. 6. The CIGS thin film is extremely dense and compact with the thickness varies in the range of 700–800 nm. Despite the thickness of the ESAVD deposited CIGS absorber is much thinner than the thickness of absorber in PVD deposited solar cells, the reduced *j*_sc_ of 29.3 mA cm^−2^ is not a main limitation on the device performance. It is demonstrated that the absorber layer is mostly composed of large grains with some grains extending over the whole thickness of the CIGS layer. Large grains generally lead to less charge recombination of the photogenerated charge carriers between grain boundaries and benefit the device performance, which meets the requirement of high efficiency solar cells. It also can be seen that there is a very thin carbon-rich layer with small grain size near the Mo back contact, which can be further optimized in the future by changing the ratio of water and ethanol in the precursor solution, in depth gradient of element, and selenization conditions.

## Discussion

The mechanism of Na doping on vacuum deposited CIGS materials has been widely studied in the past, and various mechanism have been explored^37, 38^. These include the creation of antisite acceptor defect Na_In/Ga_ by the incorporation of Na on In/Ga, neutralization of donor type defect V_Se_, and elimination of compensating antisite donor defect In/Ga_Cu_ by substituting it by Na_Cu_. The typical optimum concentration of Na in CIGS is circa. 0.1 at%, which would vary a little with different batch of glass and different CIGS deposition/selenization procedure. It is also widely accepted that Na has a low solubility in CIGS, thus Na predominately resides at grain boundaries and free surfaces. In our work, low cost and non-vacuum ESAVD method was used to deposit CIGS absorber, and Cu-poor (Cu/In + Ga = 0.76) CIGS thin films was intentionally deposited to avoid the toxic KCN treatment procedure. The CIGS absorber deposited from non-vacuum process in air normally contains more defect and shows lower efficiency (with the best efficiency around 17%) as compared with the efficiency (above 23%) of device using vacuum sputtering/thermal co-evaporation method. In addition, the lower Cu/In + Ga ratio is supposed to lead to a higher resistivity, higher carrier concentration, and higher deep level defects of the absorber^39^. Therefore, a simple dipping process was used for Na treatment of the absorber to passivate the defects at grain boundaries and improve the solar cell performance. SEM and XRD data demonstrated in the above results have shown the increased grain size and the stronger (112) crystal orientation of CIGS after Na incorporation. As compared with the mechanism published for Na doping in vacuum deposited CIGS absorber, we proposed a mechanism for the Na treating for our non-vacuum process. Na ions were absorbed at the surface and grain boundaries of CIGS absorber through the dipping process. During the selenization of the CIGS absorber, Na might be incorporated into In or Ga lattice site (replace donor defect In/Ga_Cu_ by Na/Na_Cu_) and changed the preferred orientation in the crystal. Annihilation of In/Ga_Cu_ led to better ordering of cations and resulted in an increase in the periodicity of (112) planes as evident from our XRD results in Fig. 1. The Na/Na_Cu_ replacement of In/Ga_Cu_ together with the elimination of the other deep defects in the CIGS absorber passivated the defects at the interface and grain boundaries, which helped to remove the recombination centres. Based on the above mechanism, V_oc_ of the device with Na treatment increased obviously together with improved FF and J_sc_ due to the decreased charge recombination centers. The best solar cell efficiency (without antireflection layer) of 10.83% has been achieved with the optimum Na treatment condition, which will accelerate the industrilization of ESAVD research into fully non-vacuum-based CIGSSe thin film solar cells and our findings will pave the way for dramatic improvements of the efficiency of other non-vacuum deposited chalcogenide films.

## Conclusions

In conclusion, Na treatment was proceeded by directly dipping the as-deposited CIGS thin films in NaCl aqueous solution followed by a high temperature selenization process. From the XRD results, it can be seen that Na-treatment leads to an increased in grain size and a strong (112) orientation of the CIGS film. Photovoltaic results from *j-V* curve demonstrated that Na treatment in NaCl solution followed by a high temperature selenization process clearly improved the device performance. The optimum Na treatment condition is achieved by dipping the CIGS absorber in 0.2 M aqueous NaCl solution for 20 min, which leads to an increase in all photovoltaic parameters of J_sc_, V_oc_, FF and η to 29.30 mA cm^−2^, 0.564 V, 65.59% and 10.83%, respectively. The same device showed improved photo-response in the long wavelength region, which indicates improved carrier collection efficiency due to a reduction of charge recombination loss deep in the CIGSSe thin films. A decreased [EQE(−1 V)/EQE(0 V)] ratio at long wavelength of the above 0.2 M NaCl post-treated device further implies the more effective charge separation between electron−hole pairs in the device. The study of efficiency distribution of ESAVD deposited CIGS solar cells with and without extra Na treatment showed that not only the average value but also the efficiency distribution of the solar cell devices were greatly improved after Na treatment.

## Electronic supplementary material

Supplementary Info
