# Supplementary material for: Effect of Sodium Treatment on the Performance of Electrostatic Spray Assisted Vapour Deposited Copper-poor Cu(In,Ga)(S,Se)2 Solar Cells
Source: Sci Rep. 2017 Jul 28;7:6788. doi: 10.1038/s41598-017-07027-9 (PMC5533725; doi:10.1038/s41598-017-07027-9)
Supplement: Supplementary file 1 — Supplementary Info [file 41598_2017_7027_MOESM1_ESM.docx]

Supporting Information

**Effect of Sodium Treatment on the Performance of Electrostatic Spray Assisted Vapour Deposited Copper-poor Cu(In,Ga)(S,Se)_2_ Solar Cells**

Mingqing Wang^‡^, Md. Anower Hossain^‡^, and Kwang-Leong Choy*

*UCL Institute for Materials Discovery, University College London,*

*Roberts Building, Malet Place, London, WC1E 7JE
 United Kingdom.*

*Corresponding author: [k.choy@ucl.ac.uk](mailto:k.choy@ucl.ac.uk)

^‡^Authors contributed equally.


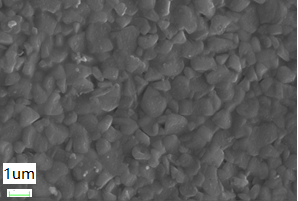

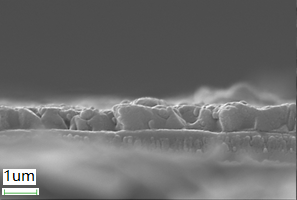


(b)

(a)


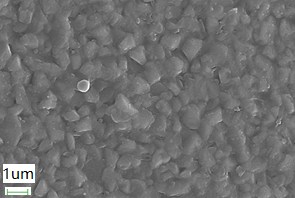

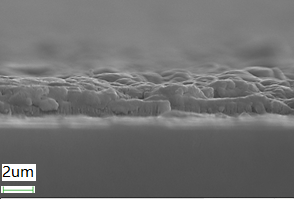


(d)

(c)

**Figure S1.** SEM top-view and cross-sectional images of the selenized CIGSSe film treated with (a, b) 0.3 M NaCl, (c, d) 0.4 M NaCl aqueous solutions for 20 minutes prior to the selenization step.
